# The Human Impact of Tropical Cyclones: a Historical Review of Events 1980-2009 and Systematic Literature Review

**DOI:** 10.1371/currents.dis.2664354a5571512063ed29d25ffbce74

**Published:** 2013-04-16

**Authors:** Shannon Doocy, Anna Dick, Amy Daniels, Thomas D. Kirsch

**Affiliations:** Johns Hopkins Bloomberg School of Public Health, Baltimore, Maryland, United States; Johns Hopkins Bloomberg School of Public Health, Baltimore, Maryland, United States; Johns Hopkins Bloomberg School of Public Health, Baltimore, Maryland, United States; Johns Hopkins University School of Medicine and Bloomberg School of Public Health, Baltimore, Maryland, United States

## Abstract

Background. Cyclones have significantly affected populations in Southeast Asia, the Western Pacific, and the Americas over the past quarter of a century. Future vulnerability to cyclones will increase due to factors including population growth, urbanization, increasing coastal settlement, and global warming. The objectives of this review were to describe the impact of cyclones on human populations in terms of mortality, injury, and displacement and, to the extent possible, identify risk factors associated with these outcomes. This is one of five reviews on the human impact of natural disasters. Methods. Data on the impact of cyclones were compiled using two methods, a historical review from 1980 to 2009 of cyclone events from multiple databases and a systematic literature review of publications ending in October 2012. Analysis included descriptive statistics and bivariate tests for associations between cyclone characteristics and mortality using Stata 11.0. Findings. There were 412,644 deaths, 290,654 injured, and 466.1 million people affected by cyclones between 1980 and 2009, and the mortality and injury burden was concentrated in less developed nations of Southeast Asia and the Western Pacific. Inconsistent reporting suggests this is an underestimate, particularly in terms of the injured and affected populations. The primary cause of cyclone-related mortality is drowning; in developed countries male gender was associated with increased mortality risk, whereas females experienced higher mortality in less developed countries. Conclusions. Additional attention to preparedness and early warning, particularly in Asia, can lessen the impact of future cyclones.

## Introduction

Tropical cyclones, also known as typhoons and hurricanes, have caused an estimated 1.33 million deaths since the beginning of the 20th century and affected more than 629 million people in this timeframe. A tropical cyclone is a non-frontal storm system that is characterized by a low pressure center, spiral rain bands and strong winds. Usually it originates over tropical or sub-tropical waters and rotates clockwise in the southern hemisphere and counter-clockwise in the northern hemisphere. Depending on their location and strength, tropical cyclones are referred to as hurricanes (western Atlantic/eastern Pacific), typhoons (western Pacific), and cyclones (southern Pacific/Indian Ocean) 1. Approximately half of tropical cyclones recorded and more than 90% of cyclone-related deaths originate in Asian waters 1. Cyclones are large organized storms with well-defined cores that begin over tropical or subtropical waters, often as a result of monsoon troughs and easterly waves 2. An average of 37 tropical storms occur each season and they range in size from 100 to more than 1,000km in diameter and are known for strong winds and bands of torrential rain that revolve around the center or eye of the storm 3. In the Eastern Pacific basin the season begins two weeks early on May 15th. The levels of intensity of these storms range from tropical depression (winds <17 meters per second), to tropical storm (winds 18-32 m/s), and cyclone (>33 m/s) 4. Once formed, cyclones maintain strength by pulling heat and moisture from warm ocean waters 3. The damage and deaths related to cyclones are the result of three major forces: winds in excess of 155 miles per hour; storm surge where the level of the sea rises as much as 10 meters and move ashore; and secondarily due to floods resulting from torrential rains. Storm surges and floods are the primary causes of death in cyclones 5.

The impacts from cyclones are concentrated in coastal areas of South and East Asia, Madagascar, the east coast of North and Central America and the Caribbean. Mortality is concentrated in Asia, economic losses follow a similar pattern; however, total economic losses are greatest in affluent countries with developed infrastructure 6. Future vulnerability to cyclones will increase due to factors including population growth, urbanization, increasing coastal settlement and changing weather patterns. The objectives of this review are to describe the impact of cyclones in terms of mortality, injury, and displacement and, to the extent possible, identify risk factors for associated with these outcomes. This is one of five reviews on the human impact of natural disasters, the others being volcanoes, floods, tsunamis, and earthquakes.

## Methods

Data on the impact of cyclones were compiled using two methods, a historical review of cyclone events and a systematic literature review of publications relating to the human impacts of cyclones.

**Historical Event Review**

A historical database of significant cyclones from 1980 to mid-2009 was created from publicly available data. Multiple data sources were sought to ensure a complete listing of events and to allow for cross checking. The two primary data sources were the Centre for Research on the Epidemiology of Disasters (CRED) International Disaster Database (EM-DAT) 7 and the National Hurricane Center (NHC)^8^ because they included information on human impacts. The events in the EM-DAT database include one or more of the following criteria: 10 or more people killed or injured; 100 people affected; declaration of a state of emergency; or a call for international assistance. The NHC database included information on all cyclones in the Atlantic, Caribbean and Gulf of Mexico.

The EM-DAT event list was downloaded in August 2009 and NHC data downloaded in February 2010. Event lists were reconciled to create a combined list of events from both data sources which were then tabulated and summarized for 1980 through 2009. See http://www.jhsph.edu/refugee/natural_disasters/_Historical_Event_Review_Overview.html for the database of tropical cyclone events. A total of 948 events were retained from EM-DAT and 331 from the NHC. For cyclone impacts reported by EM-DAT, zeroes were treated as missing values because they were used as placeholders and their inclusion in the analysis could contribute to the under estimation of tsunami impacts.

To assess risk factors for cyclone-related mortality the following categories were used: none (0 deaths), low (1-9 deaths), medium (10-99 deaths) and high (≥100 deaths). Bivariate tests for association were performed using chi-square for categorical and ANOVA for continuous measures. All covariates, with the exception of World Bank developmental level which was highly correlated with per capita GDP were subsequently included in a multinomial logistic regression model to assess relative risk of mortality at a given level as compared to events with no deaths. Analyses were performed using Stata Statistical Software, Version 11.0 9.

**Systematic Literature Review**

Key word searches in MEDLINE (Ovid Technologies, humans), EMBASE (Elsevier, B.V., humans), SCOPUS (Elsevier B.V., humans), and Web of Knowledge/Web of Science (Thomson Reuters) were performed to identify articles published in July 2007 or earlier that described natural hazards and their impact on human populations. Following the systematic review, a further search was conducted to identify relevant articles published through October 2012. One search was done for all the five natural hazards described in this set of papers. This paper describes the results for cyclones. The systematic review is reported according to the PRISMA guidelines. Key words used included *natural hazard(s), natural disaster(s), volcano(es), volcanic, volcanic eruption, seismic event, earthquake(s), cyclone(s), typhoon(s), hurricane(s), tropical storm(s), flood(s), flooding, mudslide(s), tsunami(s), and tidal wave(s).* Key words included for impact on humans were *affected, damage(d), injury, injuries, injured, displaced, displacement, refugees, homeless, wounded, wound(s), death(s), mortality, casualty, casualties, killed, died, fatality, fatalities* and had to be used in either the title, abstract or as a subject heading/key word. The search resulted in 2,747 articles from MEDLINE, 3,763 articles from EMBASE, 5,219 articles from SCOPUS, and 2,285 articles from ISI Web of Knowledge. Results from the four databases were combined and duplicates were excluded to yield a total of 9,958 articles.

Title screening was performed to identify articles that were unrelated to natural disasters or human populations. Each title was screened by two independent reviewers and was retained if either or both reviewers established that inclusion criteria were met. To ensure consistent interpretation of inclusion criteria, percent agreement was assessed across reviewers for a small sample of articles, and title screening began after 80% agreement on inclusion was achieved. A total of 4,873 articles were retained for abstract review. Articles were excluded if they met one or more of the following criteria: language other than English; editorial or opinion letter without research; not related to human populations; individual case report/study; focus on responders; and not related to human or environmental vulnerabilities or impacts of hazards. Each abstract was then screened by two reviewers and retained if either or both established that inclusion criteria were met. Included abstracts were coded for event type, timeframe, region, subject of focus, and vulnerable population focus. A total of 558 cyclone articles were retained for article review; 193 articles focusing on the impacts of cyclones on human populations in terms of mortality, injury, and displacement were prioritized for abstraction. Upon full review, 49 articles were retained including 48 that underwent dual review, standard data abstraction and one that was identified as a review article (Figure 1). The additional review then identified eleven articles through October 2012 that met the inclusion criteria for abstraction in the mortality and injury review. A summary of the final 58 abstracted articles is presented in Table 1.

**Overview of the systematic literature review process for cyclones:**
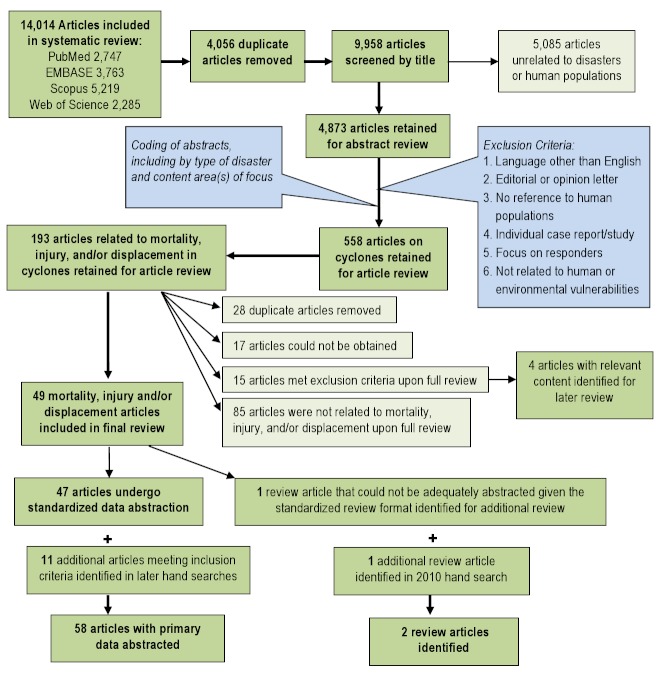

**Table 1: Articles with primary data included in the cyclone systematic literature review relating to mortality, injury, and displacement (abstracted, n=58, NR= Not Reported):** 

**Publication**	**Event(s)**	**Study Summary**	**Mortality **(n=35)	**Injury **(n=27)	**Displacement **(n=6)
Mahajani, 1975^11^	Cyclone Tracy, 1974	Post-cyclone Injury management	NR	x	NR
Longmire, 1984^12^	Hurricane Frederic, 1979	Review of injury frequency before and after the hurricane	NR	x	NR
MMWR, 1986^13^	1985Hurricanes Elena & Gloria,	Analysis of hurricane-related emergency room visits resulting in Mississippi, Rhode Island, and Connecticut	x	x	NR
Siddique, 1987^14^	Bangladesh Cyclone, 1985	Examines of risk factors for mortality among island populations	x	NR	x
Longmire, 1988^15^	Hurricane Elena, 1985	Review of injury type and severity	NR	x	NR
MMWR, 1989^16^	Hurricane Hugo, 1989	Assesses cause of death and factors associated with mortality in Puerto Rico.	x	NR	NR
MMWR, 1989^17^	Hurricane Hugo, 1989	Assesses causes of death and factors associated with mortality in South Carolina.	x	NR	NR
Philen, 1990^18^	Hurricane Hugo, 1989	Assesses mortality factors from deaths related to Hurricane Hugo.	x	NR	NR
MMWR, 1992^19^	Hurricane Andrew, 1992	Examines mortality factors in deaths reported by medical examiners in southern Florida	x	NR	NR
Rahman, 1993^20^	Bangladesh Cyclone, 1991	Evaluation of the health effects of the cyclone and tidal wave in Bangladesh.	x	NR	NR
Bern, 1992^21^	Bangladesh Cyclone, 1991	Characterizes factors associated with cyclone-related mortality and identifies prevention strategies	x	NR	NR
Chowdhury, 1993^22^	Bangladesh Cyclone, 1991	Examines mortality following 1991 cyclone and effects of cyclone preparedness	x	NR	NR
Lee, 1993^23^	Hurricane Andrew, 1992	Assesses injuries and illnesses among care seekers at health care facilities	NR	x	NR
Brewer, 1994^24^	Hurricane Hugo, 1989	Describes public health impact on inland areas of North Carolina	x	x	NR
McNabb, 1995^25^	Hurricane Andrew, 1992	Characterizes hurricane related injury and morbidity in Louisiana	x	x	NR
Combs, 1996^26^	Hurricane Andrew, 1992	Describes hurricane related population-based mortality rates	x	NR	NR
Hendrickson, 1996^27^	Hurricane Iniki, 1992	Examines hurricane-related mortality risk	x	NR	NR
Lew, 1996^28^	Hurricane Andrew, 1992	Examines damage, mortality, and displacement in Dade County, Florida	x	NR	NR
MMWR, 1996^29^	Marilyn & Opal, 1995	Injuries and health needs of affected communities in Virgin Islands, Florida, Louisiana and Georgia	NR	x	NR
MMWR, 1996^30^	Marilyn & Opal, 1995	Summarizes and characterizes hurricane-attributed deaths in Florida and US Virgin Islands	x	NR	NR
Smith, 1996^31^	Hurricane Andrew, 1992	Examines demographics effects in Dade County Florida	NR	NR	x
Hendrickson, 1997^32^	Hurricane Iniki, 1992	Uses medical chart data to characterize hurricane related increases in injuries and morbidity	NR	x	NR
MMWR, 1998^33^	Hurricane Georges, 1998	Describe deaths indirectly caused by the hurricane	x	NR	NR
MMWR, 2000^34^	Hurricane Floyd, 1999	Monitoring of illness, injury and death related to the hurricane and subsequent flooding	x	x	NR
Guill, 2001^35^	Hurricane Mitch, 1998	Assesses the impact of Hurricane Mitch on a small Honduran community	x	NR	NR
O’Hare, 2001^36^	Hurricane 07B, India, 1996	Spatial analysis of destruction caused by Hurricane 07B	x	NR	NR
Waring, 2002^37^	Tropical Storm Allison, 2001	Assesses health and medical needs of the affected population	NR	x	NR
Keenan, 2004^38^	Hurricane Hugo, 1999	Assessment of the post-hurricane incidence of traumatic brain injury in children	NR	x	NR
MMWR, 2004^39^	Hurricane Charley, 2004	Assesses causes of and factors with associated with mortality	x	NR	NR
Gagnon, 2005^40^	Hurricane Isabel, 2003	Assesses post-event injuries and injury prevention strategies	NR	x	NR
MMWR, 2005^41^	Hurricane Katrina, 2005	Documents facility-based surveillance efforts of post-hurricane effects	NR	x	NR
MMWR, 2005^42^	2004 Florida hurricanes (4)	Examines demographic and epidemiologic risk factors for hurricane outcomes	NR	x	NR
Smith, 2005^43^	Hurricane Isabel, 2003	Hurricane-related emergency department visits and storm impact on hospital admission rates	NR	x	NR
Waring, 2005^44^	Tropical Storm Allison, 2001	Utility of geographic information systems (GIS) in rapid epidemiological assessments	NR	x	NR
Brodie, 2006^45^	Hurricane Katrina, 2005	Examines demographics and health needs of evacuees in Houston area shelters	NR	x	NR
Jani, 2006^46^	Hurricane Isabel, 2003	Analysis of mortality to identify modifiable risk factors and injury prevention measures.	x	NR	NR
MMWR, 2006^47^	2004-5 Florida hurricanes (8)	Assessment of carbon monoxide poisonings reported to Florida Poison Control	NR	x	NR
MMWR, 2006^48^	Hurricane Katrina, 2005	Review county level mortality data to characterize causes of death and storm impact.	x	NR	NR
MMWR, 2006^49^	Hurricane Katrina, 2005	Describes carbon monoxide incidents and risk factors	x	NR	NR
MMWR, 2006^50^	Hurricane Katrina, 2005	Describes effectiveness of post-hurricane surveillance activities in three counties of Mississippi	x	x	NR
MMWR, 2006^51^	Hurricane Katrina, 2005	Post-hurricane surveillance of patient-specific data on injury and morbidity in greater New Orleans	x	x	NR
MMWR, 2006^52^	Hurricane Katrina, 2005	Rapid assessment of clinical care needs, public health services, and housing assistance for San Antonio evacuees	NR	NR	x
Sullivent, 2006^53^	Hurricane Katrina, 2005	Documents hurricane-related causes of injury using an active surveillance system	NR	x	NR
Vest, 2006^54^	Hurricane Katrina, 2005	Describes the prevalence of acute signs and symptoms, chronic conditions, and risk factors those in shelters	NR	NR	x
DeSalvo, 2007^55^	Hurricane Katrina, 2005	Examine post-Katrina rates and predictors of PTSD symptoms in New Orleans residents	NR	NR	x
Ghosh, 2007^56^	Hurricane Katrina, 2005	Needs assessment of the Katrina-displaced population arriving in Denver	NR	NR	x
Sharkey, 2007^57^	Hurricane Katrina, 2005	Epidemiologic review for risk factors for mortality in Hurricane Katrina	x	NR	NR
Brunkard, 2008^58^	Hurricane Katrina, 2005	Review of Hurricane Katrina deaths and risk factors in Louisiana	x	NR	NR
Eavey, 2008^59^	Hurricane Katrina, 2005	Comparison of pre- and post- Katrina mortality rates and causes	x	NR	NR
Ragan, 2008^60^	Florida, 2004-05	Mortality surveillance for eight Florida hurricanes occurring in 2004 and 2005	x	NR	NR
Das, 2009^61^	1999 cyclone in Orissa, India	Mangrove ecosystems and mortality reduction in cyclones	x	NR	NR
Shen, 2009^62^	China, 2006	Risk factors for injury during Typhoon Saomei	NR	x	NR
Uscher-Pines, 2009^63^	Hurricane Katrina, 2005	Injury and displacement among older adults following Hurricane Katrina	NR	x	x
Kanter, 2010^64^	Hurricane Katrina, 2005	Child mortality following Hurricane Katrina	x	NR	NR
Kim, 2010^65^	Cyclone Nargis, 2008	Injury and illness among Burmese patients presenting for care following Cyclone Nargis	NR	x	NR
Norris, 2010^66^	Hurricane Ike, 2008	Prevalence of disaster related illness and injury related to Hurricane Ike	NR	x	NR
Faul, 2011^79^	Hurricane Katrina 2005	Review of injuries that presented at the Houstan, Texas, Reliant Park clinic.	NR	x	NR
Zane, 2011^80^	Hurricane Ike,Texas, 2008	CDC Surveillance data on mortality in hurricane Ike	x	NR	NR

## Results

**Historical Event Review**

During the 30-year observation period (1980-2009), 1,080 cyclones were recorded with an average of 32 (range 16-66) annually. The number of events reported annually by NHC and EM-DAT increased over time as did the total number of events; NHC reported a lower number of events because their focus area is the Americas (Figure 2). Both the frequency of cyclones and affected population size increased over time; cyclone related mortality did not follow a similar trend and mortality peaks were associated with infrequent high-impact events such as cyclone Gorky (Bangladesh, 1991) and cyclone Nargis (Myanmar, 2008) (Figure 3).

**Tropical cyclone reporting frequency, 1980 -2009 (n=1080):**
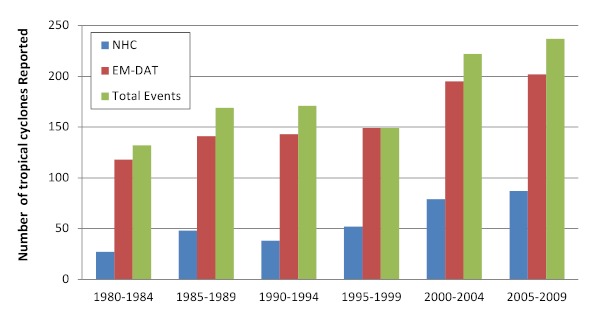

**Tropical cyclones and their affects on human populations:**
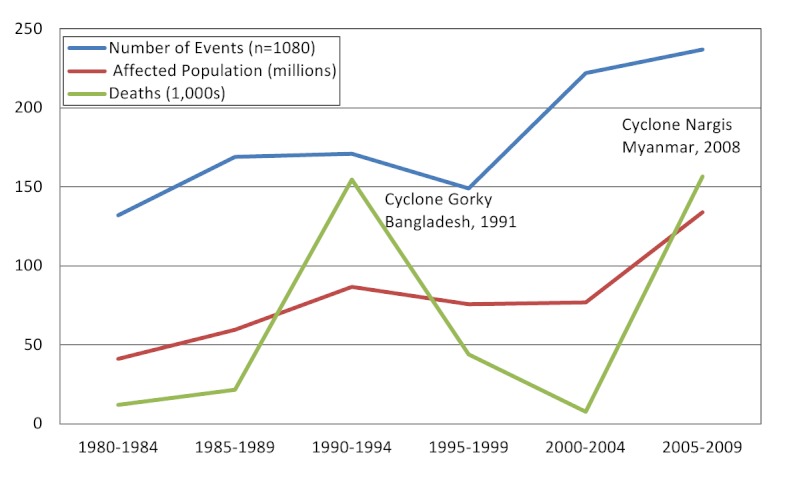

By decade, 42,5% (n=459) of events occurred in the 2000s, 29.6% (n=320) in the 1990s, and 27.9% (n=301) in the 1980s. The impact of cyclone events across regions is summarized in Figure 4. The World Health Organization regions of the Western Pacific (WPRO) and the Americas (AMRO) accounted for more than 80% of all reported events. The mortality was greatest in the SEARO regions while only 8% of deaths occurred in the AMRO region despite accounting for 37% of all events occurring there. Although the SEARO region accounted for only 9% of all events, it had 53% of the affected population and 80% of all deaths.

**Cyclones and their impact on human populations by region, 1980-2009*:**
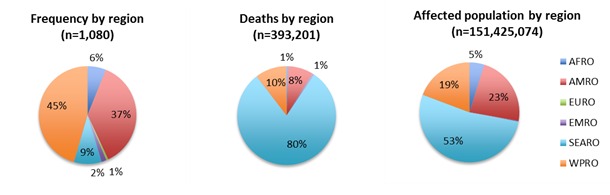
*Regions as defined by the World Health Organization

The overall impact of cyclones on human populations is summarized in Table 2. An estimated 466.1 million people were reported to be affected by cyclones between 1980 and 2009, including 20.1 million that were rendered homeless. These figures likely substantially underestimate the true impact because estimates of the total affected population and the homeless population were reported in 78.7% (n=799) and 26.2% (n=283) of events, respectively. The distribution of the affected population was highly skewed: when reported on average 592,830 people affected per storm, but the calculated median was only 20,000 per event. Monetary damages were reported by EM-DAT in 15.4% of events and evacuation was reported by NHC in only 2.3% events so these outcomes were not assessed because of insufficient reporting.

**Table 2: Summary measures for the impact of cyclones on human populations, 1980-2009 (n=1,080):** *Best estimate figures are based on the highest reported number of deaths or injuries in an event from either EM-DAT or NHC; homeless and total affected populations are reported only by EM-DAT.

***Cumulative Impact of Tropical Cyclones, 1980-2009***						
***Human Consequence***	**# of Events w/ information reported(%)**				Best Estimate *	
Deaths	1049** (97.1%)				412,644	
Injuries	340 (31.56%)				290,654	
Homeless	267 (24.7%)				20,160,878	
Total Affected	801 (74.2%)				466,098,192	
***Reporting by Source and Average Outcomes per Cyclone, 1980-2009***						
***Human Consequence***		# of Events** (%)		**Median**	**Mean **	**Range**
***Deaths, all events***		**1049**	**97.10%**	**8**	**346**	**0-138,866**
Reported by EM-DAT		925	85.60%	11	433	0-138,866
Reported by NHC		292	-27.30%	3	69	0-5,677
***Events with deaths***		**965**	**89.40%**	**14**	**430**	**1-138,866**
Reported by EM-DAT		860	79.60%	15	483	1-138,866
Reported by NHC		181	16.80%	5	87	1-5,677
***Events with Injuries***		**397**	**36.80%**	**46**	**775**	**1-138,849**
Reported by EM-DAT		338	33.90%	56	834	1-138,849
Reported by NHC		30	32.40%	3	309	1-7,242
**Homeless, all events**		**283**	**26.20%**	**5,000**	**77,907**	**25-5,432,375**
**Total Affected, all events**		**799******	**78.70%**	**20,000**	**592,830**	**2-29,622,000**

*Mortality and Injury. * Mortality data had the most complete reporting. When the two sources were combined, information on deaths was reported in 97.1% of events and deaths occurred in 89.4% of cyclones between 1980 and 2009. Overall, 412,644 deaths were reported in the historical event review, the vast majority from EM-DAT. For cyclones where mortality was reported, there was a median of 14 deaths per event (mean = 430, 5% trimmed mean= 41, range 1-138,849) when using the highest reported death toll. The two deadliest storms, Cyclone Gorky (Bangladesh, 1991; 138,866 deaths) and Cyclone Nargis (Myanmar, 2008; 138,366 deaths) accounted for two-thirds of cyclone deaths between 1980 and 2009. The next order of high mortality events with 10,000-15,000 deaths included Hurricane Mitch (Honduras, 1998) and a cyclone in Bangladesh (1985) and there were 16 events with 1,000-9,999 deaths over the 30 years. In total, the 1.9% (n=20) of events with mortality >1000 accounted for 83.9% of all cyclone deaths, suggesting that cyclone mortality is concentrated in infrequent and extreme events.

Injury data were available in 397 (36.8%) events, with a total of 290,654 cyclone-related injuries documented. When reported, there was a median of 46 injuries per storm (mean=775, 5% trimmed mean=200, range 1-138,849) when the highest reported figure was used. To estimate the total number of injuries, it was presumed that injuries occurred in events with reported deaths. There were 965 cyclones with reported fatalities. When the median and 5% trimmed mean for injuries were applied to the remaining 568 events with fatalities but no injury reporting, it was estimated that between 28,400 and 113,600 unreported cyclone related injuries may have occurred between 1980 and 2009.

Ordinal logistic regression was used to assess country-level characteristics associated with storm mortality categories (Table 3). All country-level variables were found to be significantly associated with mortality. The mean GDP per capita was $13,191 (SD 17,709, range 1433-99,383) and the mean Gini index score 10, which is a measure of equality, was 41.3 (SD 7.3, range 24.9-64.3). When assessed by mortality category, the highest per capita GDP occurred in the no deaths category while the lowest was observed in the ≥100 death category (p<.001), suggesting that the cyclone mortality risk is concentrated in lower income countries.

**Table 3. Storm mortality by select country and event characteristics (N = 1,080):** 

**Characteristic**	**0 deaths** ** (*n* = 172) **	**1-9 deaths ** **(*n* = 383) **	**10-99 deaths (*n* = 370) **	**≥100 deaths (*n* = 155) **
**Decade, n (%)**				
1980	43 (25.0%)	85 (22.2%)	109 (29.5%)	64 (41.3%)
1990	48 (27.9%)	99 (25.8%)	117 (31.6%)	56 (36.1%)
2000	81 (47.1%)	199 (51.9%)	144 (38.9%)	35 (22.6%)
**WHO Region, n (%)**				
Africa	7 (4.1%)	23 (8.2%)	28 (7.6%)	8 (5.2%)
Americas	105 (61.0%)	198 (51.7%)	95 (25.7%)	26 (16.8%)
Europe / E. Mediterranean	6 (3.5%)	10 (2.6%)	6 (1.6%)	4 (2.6%)
South East Asian	8 (4.7%)	13 (4.6%)	46 (12.4%)	34 (21.9%)
Western Pacific	57 (33.1%)	147 (38.4%)	198 (53.5%)	83 (53.5%)
**GINI Index, mean (SD)**	0 (0)	41.6 (7.7)	40.4 (7.6)	41.2 (6.7)
**GDP per capita, mean (SD)**	0 (0)	14,612 (16,653)	9,532 (15,012)	15,199 (20,057)

Relative risk ratios for cyclone mortality from the final multinomial logistic regression model, using events with no deaths as the reference category, are presented in Table 4. Per capita GDP, WHO region and event decade were significantly associated with excess mortality, in particular for the mid- and high level mortality categories (10-99 deaths and ≥100 deaths, respectively). The proportion of events with mid- to high mortality levels decreased in the 1980s and 1990s, but during the 2000s, the relative risk ratios of mid- and high level mortality events were statistically similar to the 1970s. The Western Pacific region, where the highest proportion of mid- and high level mortality events occur, was used as the reference category for regional comparisons. Relative risk ratios for mid-level mortality events were significantly lower in the European/Eastern Mediterranean, Americas, and Southeast Asia regions as compared to the Western Pacific. Relative risk ratios for high level mortality events were statistically similar to the Western Pacific for all regions except the Americas with significantly lower risk. No significant relationship between GINI index and mortality risk was observed whereas GDP was inversely associated with risk of high mortality events.

**Table 4: Multinomial logistic regression results for mortality in tropical cyclones*:** *Model Statistics: N=953, chi-square p-value

	**1-9 deaths vs. no deaths**		**10-99 deaths vs. no deaths**		**≥100 deaths vs. no deaths**	
	RRR (95%CI)	p-value	RRR (95%CI)	p-value	RRR (95% CI)	p-value
**Decade**						
1980	*Reference*		*Reference*		*Reference*	
1990	2.15 (0.89-5.18)	0.087	2.89 (1.18-7.03)	0.019	6.06 (1.95-18.72)	0.002
2000	1.93 (0.92-4.27)	0.098	1.31 (0.54-2.66)	0.597	1.03 (0.34-.3.06)	0.937
**WHO Region**						
Western Pacific	*Reference*		*Reference*		*Reference*	
Africa	1.04 (.11-1.97))	0.029	0.68 (0.32-1.44)	0.317	1.93 (0.73-5.09)	0.182
Americas	0.59 (.25-1.13)	0.054	0.38 (0.18-.82)	0.991	1.02 (0-1.09)	0.989
Europe / E. Mediterranean	0.67 (.14-1.26)	0.025	.25 (0.05-1.13)	0.073	1.79 (0.41-7.84)	0.437
South East Asia	0.75 (.16-1.19)	0.006	1.41 (0.49-4.04)	0.519	2.13 (0.80-5.66)	0.003
**Gini Index**	1.01 (.97-1.06)	0.203	0.97 (0.92-1.02)	0.21	0.97 (0.91-1.04)	0.382
**GDP per capita**	0.999 (.999-1.0)	0.936	0.999 (.999-.999)	<.001	0.999 (.999-.999)	<.001

**Systematic Literature Review**

*Mortality.* Among articles meeting inclusion criteria for full review (n=56), 34 reported mortality data including 16 that provided information on direct or indirect causes of death (Table 5) and 10 that reported sex-specific mortality counts or risk (Table 6) 11 ^-^ 66 ^,^ 80. Most articles provided some information about the distribution of deaths across population subgroups or an individual’s location at the time of the event; with one exception, all articles reported on hurricane impact in the United States. When aggregated, 54% of US hurricane deaths were classified as direct deaths and 43% as indirect deaths. Among direct deaths, drowning was the most common cause of death, accounting for 59% of direct deaths followed by trauma, which accounted for 39% of direct deaths. Among indirect deaths cause of death was less frequently reported however trauma was the most common cause of indirect death followed by motor vehicle accidents, carbon monoxide poisoning, fires or burns, and electrocution. When examined by sex, an increased mortality risk among men was observed in the eight of the ten studies that reported deaths by sex; males accounted for 59.4% of US hurricane deaths reported (Table 6). Studies that reported sex-specific mortality in the 1991 Bangladesh cyclone observed a higher mortality rates among females (71/1000) compared to males (15/1000) 20 ^,^ 21 ^,^ 22. Age was associated with increased mortality risk in numerous studies where both children 20 ^,^ 21 ^,^ 22, and older adults 22 ^,^ 57 ^,^ 58 ^,^ 60, experienced disproportionate mortality. Other risk factors for mortality included residence type 22 ^,^ 35, not reaching shelter 14 ^,^ 21, geographic location 20 ^,^ 36, race 57 ^,^ 58, flood level 57, and deforestation 28 ^,^ 61.

**Table 5: Primary research articles describing cyclone related deaths by cause and timeframe (N=17):** *direct deaths were assumed to have occurred during the event; **trauma includes blunt and penetrating trauma, crush injuries, and deaths from falling objects/debris; ***excluded from mortality totals to avoid double counting of deaths report in other sources

**Event and Reporting Information**			**Total Deaths**	**Direct Deaths**		**Indirect Deaths**			**Mortality Timeframe**							
Publication	Storm	Data Source(s)		N	By Cause	N	By Cause		Pre-	During		Post		NR		
MMWR, 1985^13^	Elena, 1985	ER Depts, Mississippi	3	0		3	2 motor vehicle accidents, 1 electrocution		0	0		0		3		
MMWR, 1989^16^	Hugo, 1989	Medical Examiner, Puerto Rico	9	2	2 drownings	7	7 electrocutions		0	3		6		0		
MMWR, 1989^17^	Hugo, 1989	Medical Examiners and Coroners, S Carolina	35	13	6 drownings, 7 blunt trauma	16	3 trauma, 13 no cause reported		0	13		16		6		
Philen, 1990***^18^	Hugo, 1989	Puerto Rico and S Carolina Medical Examiners & MMWRs	38	15	Not reported	23	Not reported		1	15		22		0		
MMWR, 1992^19^	Andrew, 1992	Florida Medical Examiner Offices	19	14	9 trauma, 4 asphyxia, 1 drowning	5	3 blunt trauma, 2 fire		0	14		3		2		
Brewer, 1994***^24^	Hugo, 1989	ER Depts, S Carolina	4	1	1 blunt trauma	3	2 vehicle accidents, 1 intracranial hemorrhage		0	0		0		4		
McNabb, 1995^25^	Andrew, 1992	ER Depts and Coroner's, Louisiana	14	6	6 drownings	8	1 motor vehicle accident, 7 no cause reported		8	6*		0		0		
Combs, 1996^26^	Andrew, 1992	Medical examiners and coroners, Florida and Louisiana	36	17	11 blunt trauma, 4 asphyxia, 2 drowning	19	3 falls, 3 fire, 3 vehicle accidents, 3 electrocutions, 2 plane crash, 2 trauma, 1 lightening strike, 1 asphyxia, 1 clean-up		2	17*		1		16		
Lew, 1996***^28^	Andrew, 1992	Medical Examiner, Dade County, Florida	17	15	8 blunt trauma, 4 asphyxiation, 2 drownings, 1 decapitation	2	2 individuals could not be reached by EMS		0	17*		0		0		
MMWR, 1996^48^	Marilyn & Opal, 1995	Medical Examiners and Coroner's, US Virgin Islands & Puerto Rico	34	18	9 blunt trauma, 8 drownings, 1 head trauma	16	7 motor vehicle accidents, 4 falling objects, 3 fires, 1 CO poisoning, 1 fall.		1	18*		7		8		
MMWR, 2000^34^	Floyd, 1999	ER Depts, N Carolina	48	36	36 drownings	12	7 motor vehicle accidents, 2 fire, 1 hypothermia, 1 fall, 1 unreported		0	36*		0		12		
MMWR, 2004^39^	Charley, 2004	Florida Medical Examiner Offices	25	9	Not reported	16	12 cause unreported, 3 CO poisoning, 1 electrocution		0	9*		0		16		
Jani, 2006^46^	Isabel, 2003	Virginia Medical Examiner's and Health Statistics	30	12	7 drowning, 5 head injuries	18	6 motor vehicle crashes, 3 head/ neck injuries, 1 trauma, 1 heart attack, 7 in power outages		0	12*		0		18		
MMWR, 2006^48^	Katrina, 2005	Florida Medical Examiner and Dept. of Forensic Science (Alabama)	19	5	3 drowning, 2 blunt trauma	14	4 vehicle accidents, 2 falling tree, 2 CO poisoning, 1 fall, 1 drowning, 1 sepsis, 1 seizure, 1 traumatic brain injury, 1 asphyxia		0	5*		0		14		
Brunkard, 2008^58^	Katrina, 2005	Federal Disaster Mortuary Op. Response Team and Louisiana coroners	986	633	387 drowning, 246 trauma or injury	338	107 heart disease, 46 other illnesses, 185 unspecified Katrina related		7	650		4		325		
Ragan, 2008^60^	Florida, 2004-05	Florida Medical Examiners Comm. and Dept. of Health	213	41	27 trauma or injury, 14 drowning	172	86 trauma or injury, 45 non-accidental, 15 CO poisoning, 9 drowning, 7 burns/inhalation		20	66		127		213		
Zane, 2011^80^	Texas, 2008	CDC Surveillence data	74	10	8 drowning, 2 Hit by falling tree limb	49	13 carbon monoxide exposure, 8 cardiovascular failure, 28 multiple causes		7	0		67		0		
**Total Number (Percent)**			**1545**	**816**	**52.8%**	**693**		**44.8%**	**45**	**746**		**231**		**633**	
**Summarized By Cause**			Drownings, n=480, 58.8%; Trauma/injury,** n=313, 38.6%; Asphyxia, n=8, 1.0%; Head/ neck injuries, n=6, 0.7%; Other/not reported, n=9, 1.1%.			Trauma/injury,** n=101, 14.6%; Vehicle accidents, n=30, 4.3%; CO poisoning, n=34, 4.9%; Fire/burns, n=17, 2.6%; Electrocution, n=12, 1.7%; Drowning, n=9, 1.3%; Head/neck injuries, n=4, 0.6%; Other, n=250, 36.1%; and Not reported, n=206, 32.0%			**2.9%**	**45.0%**		**13.9%**			**38.2%**

**Table 6: Primary research articles describing cyclone related deaths sex (N=11):** 

Source	Storm	Location(s)	Gender most at risk	Deaths by Sex		Summary of gender-related mortality findings
				Males	Females	
Bern, 1992^21^	Bangladesh, 1991	Bangladesh	Female			Mortality among females was higher than males for all age groups; for females, mortality increased with age.
Chowdury, 1993^22^	Bangladesh, 1991	Bangladesh	Female			The female mortality rate was 71/1000 as compared to 15/1000 among males ages 20-44. Death rates were higher among females, and this was more pronounced in the young and old.
Combs, 1996^26^	Andrew, 1992	Florida and Louisiana	Male	40	15	73% (40/55) of deaths were among males. Male and female mortality rates in Florida were 18.8 and 7.3 per 1,000,000, respectively. Male and female mortality rates in Louisiana were 5.8 and 1.2 per 1,000,000, respectively.
MMWR, 1996^30^	Marilyn and Opal, 1995	Puerto Rico, Florida, N Carolina, Alabama, Georgia	Male	21	6	78% (21/27) of the deceased were male.
MMWR, 2000^34^	Floyd, 1999	North Carolina	Male	38	14	73% (38/52) of the deceased were male.
MMWR, 2004^39^	Charley, 2004	Florida	Male	24	7	77% (24/31) of the deceased were male.
Jani, 2006^46^	Isabel, 2003	Virginia	Male	24	8	77% (24/32) of the deceased were male.
Sharkey, 2007^57^	Katrina, 2005	Louisiana	Male			Males accounted for 65% of non-elderly deaths and 48% of the nonelderly population; 47% of elderly deaths were among males who accounted for 38% of the elderly population
Brunkard, 2008^58^	Katrina, 2005	Louisiana	Male	512	459	53% (512/971) of the deceased were male.
Ragan, 2008^60^	2004 & 2005 hurricanes	Florida	Male	162	51	76% (162/213) of deceased were male.
Zane, 2011^80^	Ike, 2008	Texas	Male	52	22	70% were male.
			**Total**	**873**	**582**	
				**60%**	**40%**	

*Injury.* Injury data were reported in 28 of the 58 articles, 15 of which provided information on injury type (Table 7). Most articles reported that the majority of injuries were minor but it was not possible to aggregate injury data due to the different study designs, reporting methods, and data sources. Lacerations, wounds, contusions, blunt trauma, animal/insect bites, and motor vehicle injuries were among the most frequent types of injuries reported. The three population based surveys estimated injury rates between 3.8 and 4.5% 37 ^,^ 62 ^,^ 66. When assessed by age, injury rates were highest among middle age adults in numerous studies 13 ^,^ 25 ^,^ 32 ^,^ 40 ^,^ 53. Males were at higher risk for injury 13 ^,^ 24 ^,^ 25 ^,^ 54; in all studies but one in the studies that reported gender 22. Location was a risk factor for injury in many reports, including administrative unit or location relative to storm path 42, within a city 24 ^,^ 25 ^,^ 44, and being outdoors 25. Race was associated with increased injury risk in several studies, however, the race most at risk for injury varied 24 ^,^ 25 ^,^ 26 ^,^ 56. Only three articles presented injury data on storms outside the United States 11 ^,^ 62 ^,^ 65, reflecting the paucity of information from less developed countries and a need for additional research in cyclone injury epidemiology in these regions.

**Table 7: Summary of Primary Research Articles with Injury Findings (n=27):** 

Publication	Event(s)	Study Type	Injuries Reported	Types of Injuries Reported	Additional Injury Findings
Mahajani, 1975^11^	Cyclone Tracy, 1974	Facility, inpatient only	145	60 lacerations (41%), 50 blunt trauma (34%), 14 spinal cord injuries/ paraplegia (10%), 6 pelvis fractures (4%), 3 penetrating wounds (2%), 3 closed abdominal injuries (2%), 2 head injuries (1%), 1 amputation (<1%).	None
Longmire, 1984^12^	Hurricane Frederic, 1979	Facility, ER visits	Not reported	Not reported	Lacerations, puncture wounds, chain saw injuries, burns, gasoline aspiration, gastrointestinal complaints, stings, and spouse abuse were found to increase following the storm.
MMWR, 1986^13^	Hurricanes Elena & Gloria, 1985	Facility, ER visits	484	Lacerations (22%), abrasion or contusion (20%), sprain (14%) and fractures (12%).	89 records were visits related to the storm, 73 were injuries. 26 of 73 patients had lacerations and 11 had fractures.
Longmire, 1988^15^	Hurricane Elena, 1985	Facility, ER visits	2623	Tables not legible	There was a significant increase in the number of patients treated for blunt trauma, chain saw injuries, and lacerations, following the storm.Top of FormBottom of Form
Lee, 1993^23^	Hurricane Andrew, 1992	Facility, ER and outpatient	Not reported	Not reported	Injuries accounted for 15.7% and 23.7% of visits at civilian and military free care sites; among service members, injuries accounted for 36.2% of visits. During the 5 weeks after the hurricane, proportional morbidity from injury decreased.
Brewer, 1994^24^	Hurricane Hugo, 1989	Facility, ER visits	1911	577 wounds (28%), 428 insect stings (21%) 279 sprains (12%), 241 contusions (12%), 177 fractures (8%), and 131 other injuries (6%), and 78 unknown (4%).	88% if hurricane diagnoses were injury related. Incidence of diagnoses varied by age, sex, race and care seeking location.
McNabb, 1995^25^	Hurricane Andrew, 1992	Facility, ER visits	375	184 cuts/lacerations/puncture wounds (49%), 49 sprain/strain/ fracture (13%), 46 contusion/ impact (12%), 24 animal/insect bite (6%), 23 falls (6%), 23 rashes (6%), 15 crush injuries (4%), 10 burns (3%), 1 electrocution (<1%), and 62 other (17%).	Injuries accounted for 86% of non-fatal events. Injury rates were highest among middle age adults (30-39 yrs) and were concentrated geographically in three parishes.
MMWR, 1996^29^	Hurricanes Marilyn and Opal, 1995	Facility, outpatient visits	234	80 lacerations/wounds (34%), 79 sprain/strain/fracture (34%), 37 motor-vehicle related injuries (16%), 38 other (16%).	Of 3265 facility visits, 1084 (33%) were storm-associated injuries involving minor wounds or musculoskeletal trauma.
Hendrickson, 1997^32^	Hurricane Iniki, 1992	Facility, ER and inpatient	1584 post-storm	865 open wounds (55%), 196 sprains (13%) 148 contusions (9%), 122 superficial wounds (8%), 83 insect/animal bites (5%), 81 fractures (5%), 29 foreign bodies (2%), 23 burns (1%), 10 head injuries (1%), and 5 poisoning (<1%).	The relative risk for injury was 6.86 (95 CI: 5.98–7.87) in the two week period after the storm as compared to the two weeks prior to the event. Injury risk increased for all age and sex groups; open wounds and foreign objects injuries had the greatest increase post-storm.
MMWR, 2000^34^	Hurricane Floyd, 1999	Facility, ER visits	~19780	Not reported	33% of ER visits (n=59,398) were injury related; soft tissue injuries accounted for 28% of ER visits (~16,631) and the majority of injuries.
Waring, 2002^37^	Tropical Storm Allison, 2001	Population based post-disaster assessment	17 households	Not reported	Injury types included abrasion/cut/puncture and animal bites; no significant difference in injury was observed between individuals from flooded and non-flooded homes.
Keenan, 2004^38^	Hurricane Hugo, 1999	Ecological	Not applicable	Not applicable	An increase in inflicted and non-inflicted traumatic brain injury was observed among young child in the 6 months following the storm.
Gagnon, 2005^40^	Hurricane Isabel, 2003	Facility, ER visits	51 attributed to the storm	Most common injuries were lower extremity fractures (21%), abrasions/sprains (16%) and rib fractures (12%).	59% of injuries were tree related; most patients had severe and multiple injuries and one-third were admitted. Males age 50-60 had the highest incidence of injury.
MMWR, 2005^41^	Hurricane Katrina, 2005	Facility, ER and outpatient	2018	716 unintentional injuries including cuts, blunt trauma, burns and environmental exposures (36%), 464 falls (23%), 311 bites/stings (15%), 145 vehicle crash injuries (7%), 42 intentional injuries (2%), 27 other toxic exposure (1%), 14 CO poision (1%) and 299 unknown (15%).	14% of visits were relief workers, 34% were residents, and 52% were unknown; relief workers were 5.8 (CI:5.0-6.8) times more like to be treated in nonhospital facilities than residents.
MMWR, 2005^42^	4 Florida hurricanes in 2004	Telephone survey	1690	Not reported	Physical injuries caused by the hurricanes were reported by 4.6% of persons in the hurricane paths and 3.8% not in the hurricane paths.
Smith, 2005^43^	Hurricane Isabel, 2003	Observational cohort, ER patients	Not reported	Not reported	Cases of major trauma decreased by 50% and minor trauma increased by 57% in the 5 day post-landfall period.
Waring, 2005^44^	Tropical Storm Allison, 2001	GIS based post-disaster assessment	Not reported	Injuries were minor; the most common injury types reported were cuts/scrapes/scratches, animal/insect bites, and blunt trauma/bruising.	Persons in flooded homes were 4.8 (CI:1.9-12.8) times more likely to be injured than those living in non-flooded homes.
Brodie, 2006^45^	Hurricane Katrina, 2005	Post-disaster survey in shelters	Not reported	Not reported	33% of evacuees with children and 29% without children were injured; 13% in each group reported serious injuries. Those who evacuated prior to the storm had a 26% injury rate as compared to 37% of those who did not evacuate.
MMWR, 2006 47	8 Florida hurricanes, 2004-05	Health facility	Not reported	Not reported	Increased number of CO poisonings and hydrocarbon fuel exposures were observed in the post-storm periods.
MMWR, 2006^50^	Hurricane Katrina, 2005	Facility, ER and outpatient	10298	Not reported	Between Sept 5-11, there 4,391 visits for injuries, including 1,324 (30%) for tetanus vaccination with no further injury description. Between Sept 12-Oct 11 (after active surveillance) there were 5,907 visits for injuries including 497 (8%) major and 5,410 (92%) minor injuries.
MMWR, 2006^51^	Hurricane Katrina, 2005	Facility, ER and outpatient	4579	2,411 unintentional injuries including cuts, blunt trauma, burns and environmental exposures (53%), 992 falls (22%), 416 vehicle crash injuries (9%), 339 animal/insect bites (7%), 89 intentional injuries (2%), 34 toxic exposure/poisoning (<1%), and 298 unknown (7%).	Residents had a higher proportion of falls and motor vehicle accidents and a lower proportion of unintentional injuries as compared to relief workers.
Sullivent, 2006^53^	Hurricane Katrina, 2005	Facility, ER and outpatient	7543	Cut/pierce/stab (20%), fall (20%), struck by/against/ crushed (11%), bite/sting (9%), and motor-vehicle crash (8%).	The leading mechanisms of injury were falls and cut/stab/pierce sounds, with a greater proportion of residents being injured as compared to relief workers; clean-up was the most common activity at the time of injury for both groups.
Shen, 2009^62^	Typhoon Saomei, China, 2006	Town census	136	71 cut/stabbed (55%), 41 blunt trauma (32%) 13 falls (10%), 3 crushed (2%) and 1 drowning (<1%).	Injury rate of 4.5%, including 7 deaths resulting from injury. Residences facing the sea, end units, non-reinforced windows/doors, and staying near a window/door or in a damaged room were associated with increased injury risk.
Uscher-Pines, 2009^63^	Hurricane Katrina, 2005	Review of medicare claims (older adults)	3870 in the year following the storm	1678 sprains/strains (43%), 1026 other fractures (27%), 980 lacerations (25%), and 186 hip fractures (5%)	Prevalence of all injury types increased post-storm; displaced storm victims were at increased risk for hip (OR 1.53, CI: 1.10-2.13) and other (OR 1.24, CI: 1.07-1.44) fractures.
Kim, 2010^65^	Cyclone Nargis, 2008	Outpatient medical record review	128	Not reported	5% of patients had trauma/injuries of which 29% were directly related to the cyclone
Norris, 2010^66^	Hurricane Ike, 2008	Population based survey	37	Not reported	Injury rate of 3.8%; risk of injury increased with damage and decreased with evacuation.
Faul, 2011^79^	Hurricane Katrina, 2005	Outpatient medical record review	1130	Injuries to the elbows/wrist/hand/finger (rate = 38.9; 95% CI = 28.3-52.2), face/trunk/shoulder/upper arm (rate = 31.8; 95% CI = 22.3-44.1), and leg/foot/toe (rate =151.2; 95% CI = 129.4-175.7).	Significantly more wound injuries to the lower extremities (rate = 13.7; 95% CI = 11.6-16) and upper limbs (rate = 6.5; 95% CI =5.1-8.2).

## Discussion

**Main Findings**

In the 30 year period between 1977 and 2009, approximately 466 million people were affected by cyclones; 20.1 million left homeless, 412,000 people died and 290,000 were injured, excluding an estimated 28,000 to 114,000 unrecorded injuries. The mortality estimate presented in this study is consistent with recent estimates in other studies 67, but the numbers injured and displaced are likely gross underestimates given the low frequency with which these figures are reported. Findings from the historical event review are also consistent with previous observations that cyclone mortality varies by region, economic development level, and event severity.Cyclone impacts were concentrated in the Asia-Pacific region and the majority of fatalities occurred in developing nations. High mortality events, with death tolls in excess of 1000, occur in less than 2% of events and more than two-thirds of all cyclone deaths between 1980 and 2009 occurred in two events (Cyclone Gorky, Bangladesh 1991 and Cyclone Nargis, Myanmar 2008). The number of cyclones and deaths increased each decade but the average number of deaths per storm decreased. Human vulnerability to cyclones will increase in future years due to population growth, urbanization, increased coastal settlement, poverty, and changing weather patterns which is associated with an increase in the number of high intensity cyclones.

A significant disparity between cyclone mortality in developing and developed nations persists, though apart from simple casualty counts there is little information available on the epidemiology of cyclone morbidity and mortality in less developed countries. This indicates a need for additional research outside of the US. The United Nations Development Program (UNDP) identified 29 developing nations and four developed nations that are at risk for cyclones 67 but 42% and 27% of cyclone deaths in the past two centuries have occurred in Bangladesh and India, respectively 68. Additionally, the majority of high-fatality storms occurred in the latter half of the 20th century though no developed nation sustained more than 1000 deaths from a cyclone in this time period^67^^,^ 69. The leading explanations for regional differences in mortality is the size of the at risk population and the capacity for pre-event evacuation. Higher population densities in the Western Pacific and South East Asia where dense settlements in low lying areas are associated with increased risk of death in from storm surge 70 ^,^ 71. A higher economic development among the countries in the Americas is associated with lower regional mortality rates because of increased capacity to evacuate. Prior to the implementation of early warning, evacuation, and shelter systems an estimated 90% of cyclone mortality was attributed to storm surge drowning 72 ^,^ 73 ^,^ 74 ^,^ 75 ^,^ 76. Improvements in forecasting, and early warning systems and in evacuation and shelter procedures, particularly in developed countries, have reduced storm-surge related mortality and increased proportional morbidity and mortality in the post-impact period 16 ^,^ 19 ^,^ 69.

**Comparison with Previous Reviews**

Findings of this review were contrary to the conclusions of other recent reviews which concluded that most storm-related mortality in developed countries occurs in the post-impact period 69. In the systematic literature review, 79% of the 946 included deaths where storm phase was reported occurred during the impact period. Direct and indirect deaths, respectively, accounted for 56% and 44% of deaths (n=1450 deaths where cause was reported) and the primary causes of death were drowning (33%), direct injuries or trauma (21%), and indirect injuries or trauma (7%). Studies that included gender breakdown for cyclone-related deaths, most of which are accounts of cyclone events in the United States, consistently reported greater proportion of male as compared to female deaths; when aggregated, males accounted for 59% of reported US hurricane deaths. In less developed countries females face a greater mortality risk 21 ^,^ 22. An increased risk of death in younger 20 ^,^ 21 ^,^ 22, and older populations 22 ^,^ 57 ^,^ 58 ^,^ 60, was also observed which is consistent with broader natural disaster mortality trends. However, it is important to note that primary research on cause and timeframe of death as well as demographic and other factors associated with increased mortality risk is limited almost completely to the United States. Future studies on the human impacts of cyclones should be focused in Southeast Asia and the Western Pacific, the regions where the majority of cyclone impacts occur.

While minimal data on cyclone-related injuries and mortality is available from less developed settings, it can be presumed that developing nations also bear the burden of cyclone-attributable injury where the frequency and severity of injuries are inversely related to degree of physical protection 69. Minor trauma is common among the injured, including lacerations, abrasions and contusions, puncture wounds, and sprains and fractures 11 ^,^ 13 ^,^ 24 ^,^ 25 ^,^ 29 ^,^ 32 ^,^ 40 ^,^ 41 ^,^ 51 ^,^ 53 ^,^ 62 ^,^ 63. The majority of those injured in cyclones can be treated on an outpatient basis and do not require sophisticated surgical or inpatient care 5 ^,^ 77. Reported causes of death and injury in more developed countries during the post-impact phase include blunt trauma, vehicle accidents, carbon monoxide poisoning, burns and fires, electrocution, and chain-saw injuries. As compared to needs for food, water, shelter, and sanitation, injuries are not usually a major public health problem in the post-impact phase 78 This suggests and that mobile field hospitals and specialized surgical teams may be ineffective responses and that non-medical relief may be a more appropriate strategy for morbidity and mortality reduction in the aftermath of cyclones 5.

**Limitations**

The availability and quality of data has likely increased and improved over time, however, in many events deaths, injuries, and affected population size are unknown or unrecorded. For most events no data were reported for injured, displaced, and affected populations, contributing to underestimation of impacts. Inconsistencies and errors were common in data from different sources that called into question the reliability of available data. In some cases inclusion criteria and definitions were not ideal which created difficulties in reconciling event lists. Challenges were encountered when modeling cyclone mortality including a non-normal distribution, which necessitated analysis with a categorical outcome. Information on 2007-2009 GDP and 2009 GINI index were used for analysis regardless of the event year, but these values may have been different for events in the 1980s. Additionally, some countries did not exist or have merged with other nations since the 1980s and many of the smaller island countries in the Caribbean are territories of European countries which necessitated the use of GDP, GINI, and development levels which may not be representative of realities in the cyclone affected area and/or time period. When combined with uncertainty in the historical record and the relative paucity of primary research focusing on cyclone impacts in heavily affected Asian region, conclusions that can be drawn about cyclones impacts on human populations are limited. Other principal limitations of the literature review are 1) that an in-depth quality analysis of all reviewed articles was not undertaken, and 2) the fact that only English language publications were included which likely contributed to incomplete coverage of studies published in other languages originating from low and middle income countries.

## Conclusions

Analysis of the impact of cyclones on human populations is challenging given the paucity of data from the most affected regions, the occasional occurrence of extreme high mortality events, and the reporting inconsistencies including both lack of standardized definitions and temporal changes in collection procedures, completeness and accuracy of data. However, even with this under-representation the impact of cyclones is huge, with 466 million people affected, 412,644 deaths and 290,654 injuries were reported as a result of cyclones between 1980 and 2009. The primary cause of cyclone-related mortality in both developed and less developed countries was storm surge drowning. In more developed countries an increased proportion of deaths and injuries were observed in the aftermath of cyclones as a result of improved early warning systems and evacuation. Male gender was associated with increased mortality risk in developed countries, whereas female gender was linked to higher mortality risk in less developed countries. Both older and younger population sub-groups also face an increased mortality risk.

Cyclones have significantly impacted populations in Southeast Asia, the Western Pacific, and the Americas regions over the past quarter of a century with less developed nations in Asia bearing the majority of the mortality and injury burden. Additional preparedness and mitigation strategies, particularly in less developed countries where the majority of cyclones occur, can lessen the impact of future events. In particular, improvements in forecasting, early warning systems, evacuation and shelter procedures, and public education on safety precautions and post-impact hazards could reduce cyclone-related morbidity and mortality in future decades.

## Competing Interests

The authors have no competing interests to declare.

## Correspondence

Shannon Doocy, Johns Hopkins Bloomberg School of Public Health, 615 N. Wolfe St, Suite E8132, Baltimore, MD 21230. Tel. 410-502-2628. Fax: 410-614-1419. Email: sdoocy@jhsph.edu.

## Appendix 1

### PRISMA Checklist

Download PDF
